# Deep Stromal Honeybee Sting Injury Causing Toxic Keratouveitis, Secondary Ocular Hypertension, and Iris Atrophy With Excellent Visual Recovery: A Case Report

**DOI:** 10.7759/cureus.114971

**Published:** 2026-08-22

**Authors:** Piyusha N Dinde, Prerna Upadhyaya, Shubha Rai

**Affiliations:** 1 Ophthalmology, Sewa Sadan Eye Hospital, Bhopal, Bhopal, IND

**Keywords:** corneal edema, corneal foreign body, honeybee sting, keratouveitis, ocular hypertension

## Abstract

Honeybee sting injuries involving the cornea are uncommon but can result in severe visual morbidity because of mechanical injury and venom-induced toxicity. A 29-year-old man presented with severe pain, redness, watering, and profound visual loss in the right eye following accidental entry of a honeybee while driving. Visual acuity was perception of light with accurate projection of rays in all four quadrants. Slit-lamp examination revealed marked corneal edema, Descemet membrane folds, and a deep stromal foreign body in the superotemporal cornea. Intraocular pressure was elevated to 42 mmHg. Urgent foreign body removal and corneal scraping were performed, followed by intensive medical management including topical antibiotics, cycloplegics, antiglaucoma therapy, and systemic medications. Serial follow-up demonstrated progressive resolution of corneal edema and inflammation. Although diffuse iris atrophy and a fixed dilated pupil developed, final visual acuity improved remarkably to 6/6 partial. Early recognition and prompt intervention can result in excellent visual outcomes despite severe initial presentation.

## Introduction

Ocular bee sting injury is a rare but potentially vision-threatening ophthalmic emergency. Although most bee stings involve the skin, ocular involvement is uncommon and may result in significant morbidity due to both direct mechanical injury from the retained stinger and the toxic and immunologic effects of bee venom. Previous reports have described a wide spectrum of ocular manifestations following hymenopteran insect stings, ranging from mild conjunctival inflammation to severe corneal and intraocular complications [1,2]. The clinical presentation is highly variable and depends on the site and depth of penetration, the quantity of venom injected, and the host inflammatory response. Manifestations may range from mild conjunctivitis and superficial keratitis to severe complications such as toxic keratitis, corneal edema, endothelial decompensation, anterior uveitis, secondary glaucoma, cataract formation, optic neuropathy, and permanent visual loss [3-5].

Honeybee venom contains biologically active substances, including melittin, phospholipase A₂, hyaluronidase, apamin, and other inflammatory peptides, which can induce corneal endothelial toxicity, stromal inflammation, and the breakdown of the blood-aqueous barrier [6]. These mechanisms contribute to the development of toxic keratopathy, persistent corneal edema, uveitis, and secondary ocular hypertension. Because of the rarity of ocular bee sting injuries, there is no standardized management protocol, particularly regarding the timing of stinger removal and the use of corticosteroids [4,7,8]. Early recognition, careful slit-lamp examination to detect retained stinger fragments, and prompt initiation of appropriate medical or surgical treatment are critical for preserving ocular integrity and achieving favorable visual outcomes [5,7].

We report a case of a deep stromal honeybee sting injury presenting with toxic keratouveitis and secondary ocular hypertension. Despite the potentially sight-threatening nature of the injury, early diagnosis and prompt intensive medical management resulted in the complete resolution of inflammation and excellent visual recovery. This case highlights the importance of early intervention and close follow-up in optimizing outcomes following this uncommon ocular emergency.

We report a case of a deep stromal honeybee sting injury presenting with toxic keratouveitis and secondary ocular hypertension. Despite the potentially sight-threatening nature of the injury, early diagnosis and prompt intensive medical management resulted in the complete resolution of inflammation and excellent visual recovery. This case highlights the importance of early intervention and close follow-up in optimizing outcomes following this uncommon ocular emergency.

## Case presentation

A 29-year-old man presented to the emergency ophthalmology department with complaints of severe pain, redness, watering, foreign body sensation, and sudden diminution of vision in the right eye following accidental entry of a honeybee while driving. On presentation, best-corrected visual acuity (BCVA) in the right eye was perception of light with accurate projection of rays, whereas the left eye had visual acuity of 6/6. Intraocular pressure measured by iCare tonometry (Vantaa, Finland) was 42 mmHg in the affected eye.

**Figure 1:**
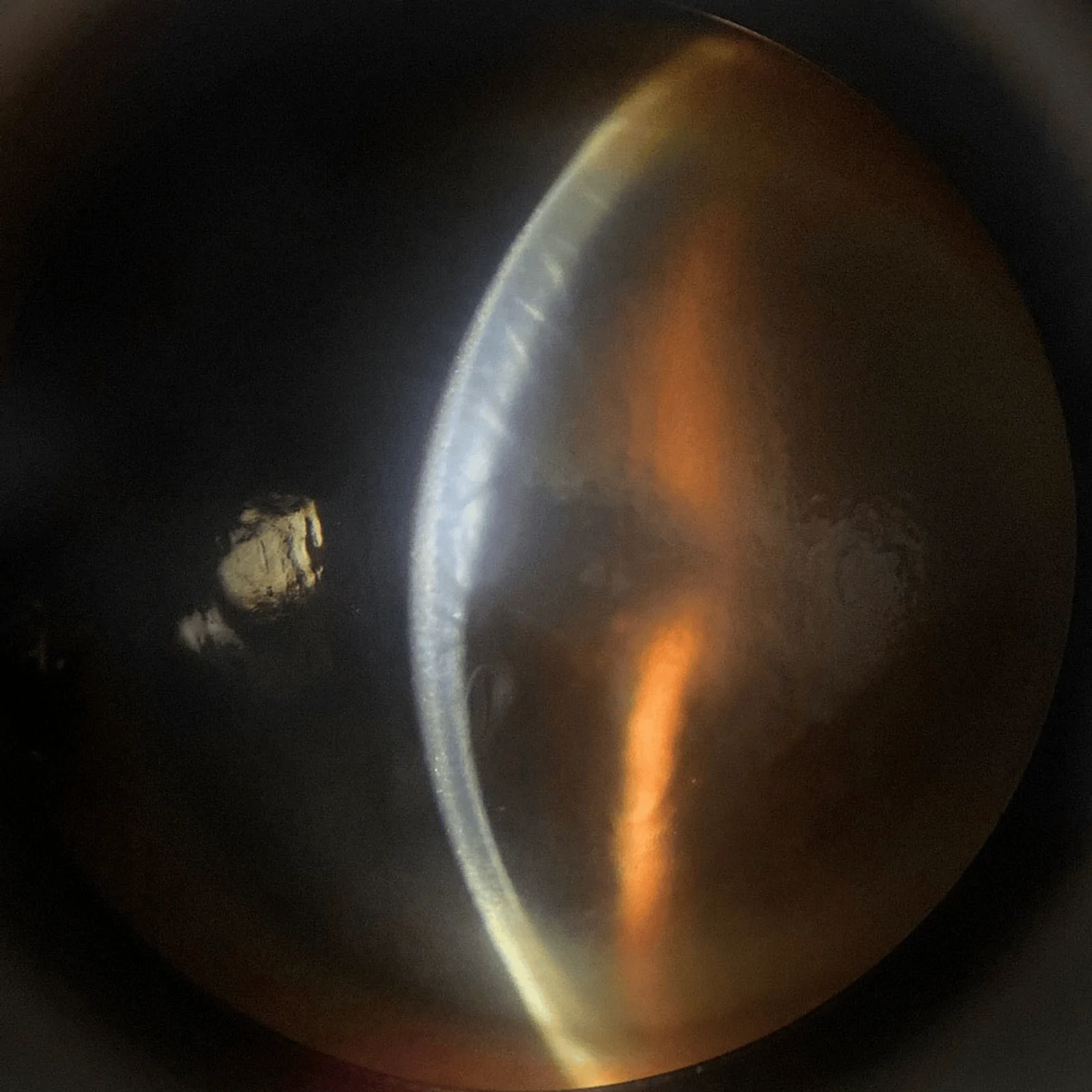
Slit-lamp image showing stromal edema post-foreign body removal (day 1)

Slit-lamp examination revealed upper lid edema, circumcorneal congestion, marked corneal edema (+3), extensive Descemet membrane folds, and a deep stromal foreign body (bee sting) located in the superotemporal cornea. The foreign body was surrounded by stromal infiltration. Visualization of the anterior chamber was significantly compromised because of dense corneal edema (Figure 1).

**Figure 2:**
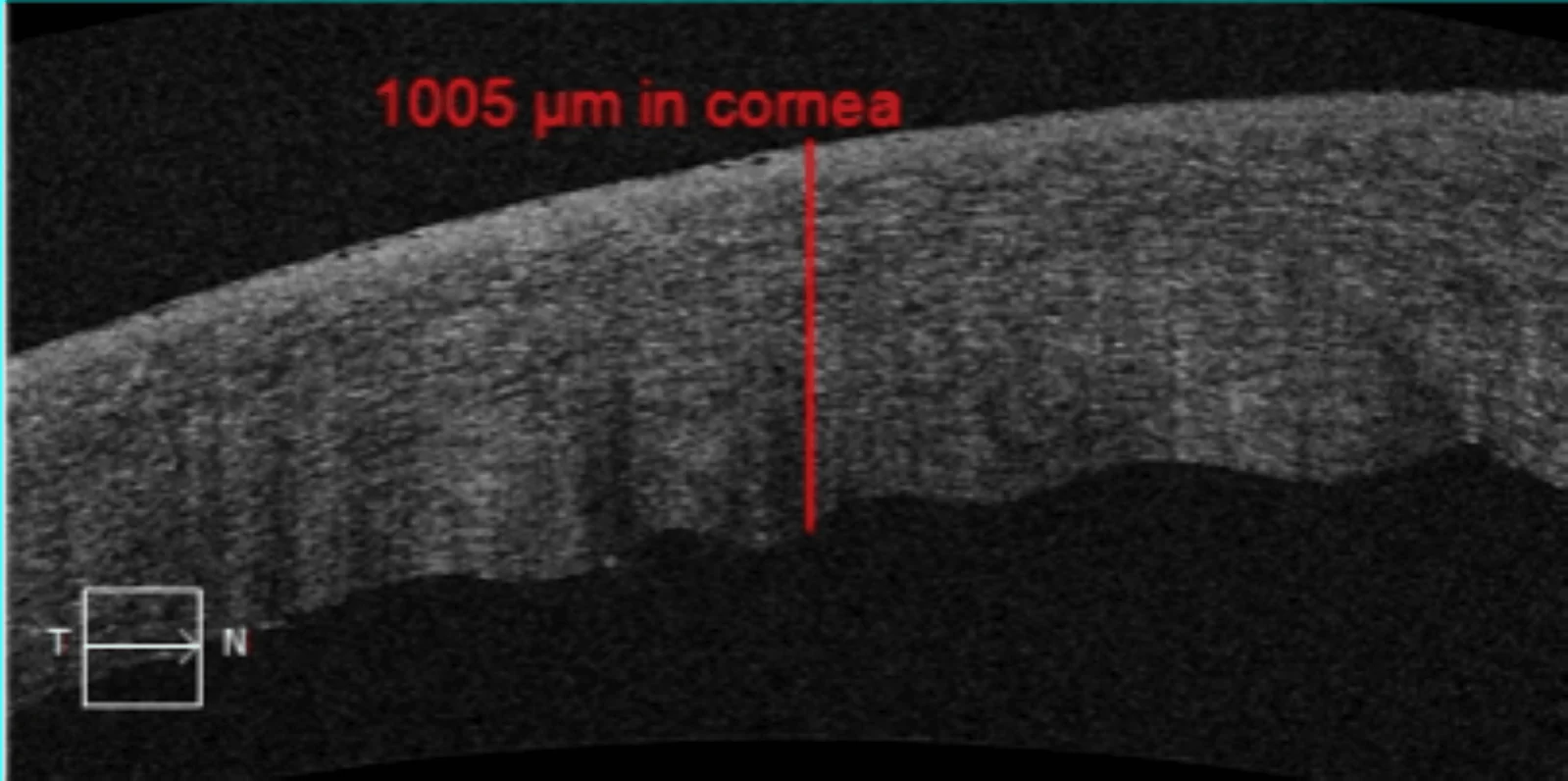
Baseline anterior segment optical coherence tomography showing marked corneal stromal edema and hyperreflective stromal tract corresponding to the retained honeybee sting foreign body

Urgent corneal scraping and deep stromal foreign body (bee sting) removal were performed under local anesthesia. Microbiological evaluation of the corneal scraping was negative for bacterial and fungal organisms. Baseline anterior segment optical coherence tomography was done to note the corneal thickness and Descemet membrane folds (Figure 2).

Post-foreign body removal procedure, the patient was treated with topical moxifloxacin 0.5% eye drops every two hours, atropine 1% eye drops three times daily, oral ciprofloxacin 500 mg twice daily, intravenous mannitol 300 mL, oral acetazolamide 250 mg, and topical antiglaucoma medication eye drop timolol 0.5% twice daily.

**Figure 3:**
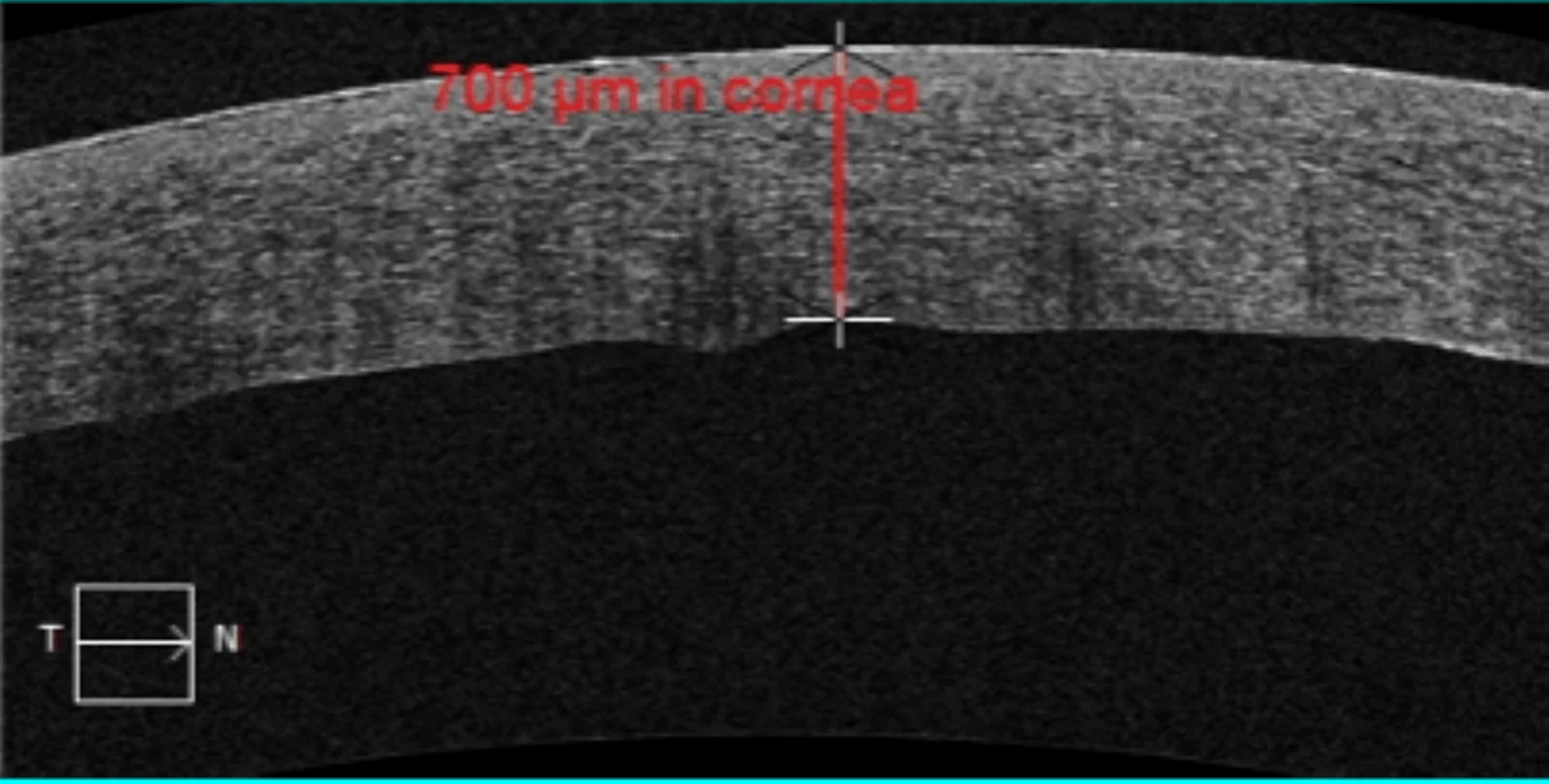
One-week postoperative anterior segment optical coherence tomography demonstrating reduction in stromal edema following foreign body removal

At the one-week follow-up, visual acuity improved to 1/60 with reduction in corneal edema and Descemet membrane folds. Anterior segment optical coherence tomography demonstrated residual stromal hyperreflectivity corresponding to the previous site of bee sting penetration with improving corneal architecture (Figure 3).

At two weeks, visual acuity improved to 2/60 with further reduction in stromal edema. Three weeks following injury, visual acuity improved to 6/36 and further to 6/18 with pinhole correction. AC flare (2+) with pigmented and non-pigmented keratic precipitates was noted. Topical low-dose steroid loteprednol, tapered from four times a day for five days, was added to medical management at two weeks post-injury, only after the infective component was ruled out.

**Figure 4:**
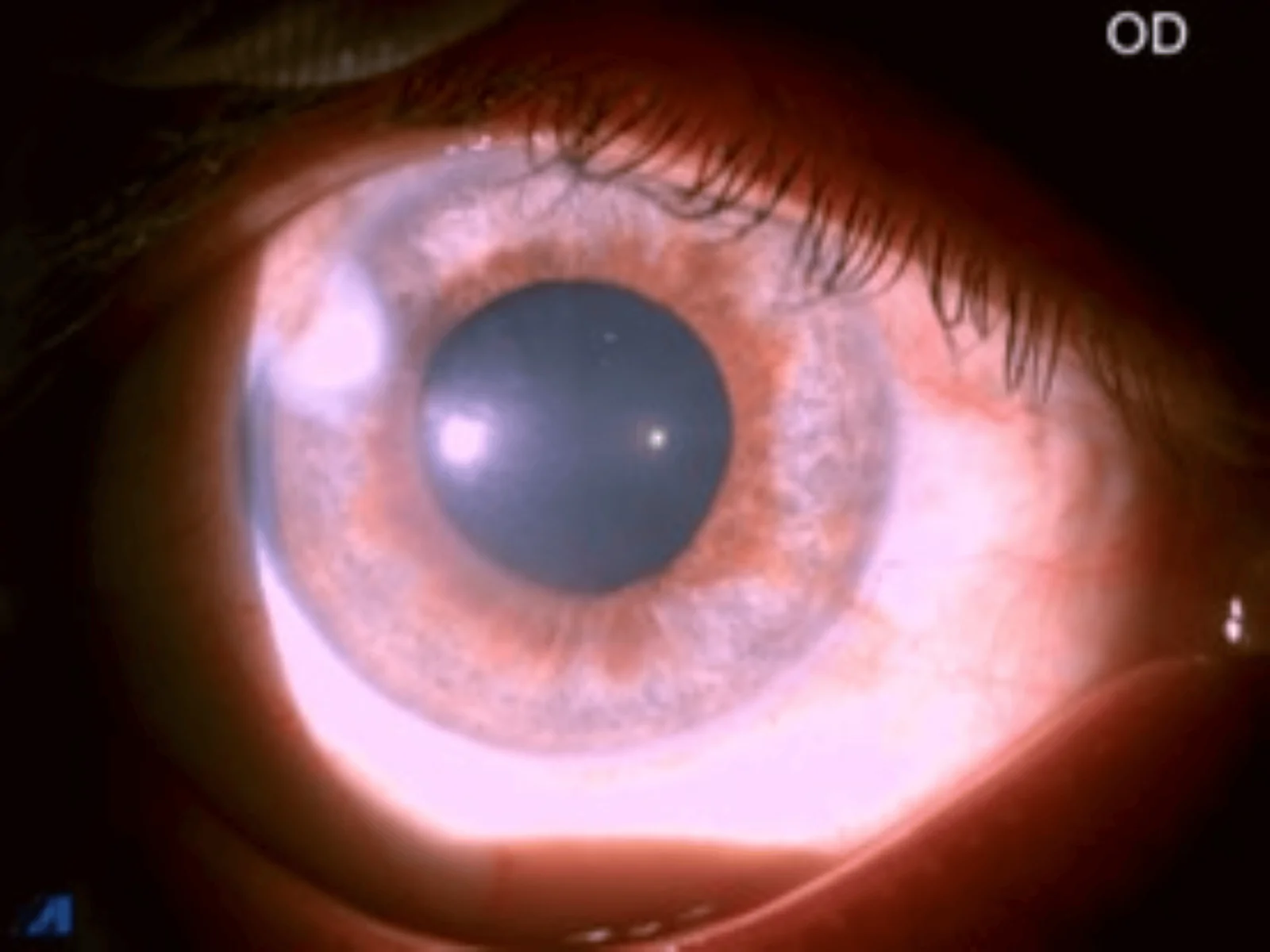
Slit-lamp photograph of the right eye demonstrating diffuse iris stromal atrophy, extensive iris depigmentation, and a persistently dilated pupil following honeybee sting injury

At the one-month follow-up, significant resolution of corneal edema was observed. Specular microscopy demonstrated gradual recovery of endothelial morphology. Slit-lamp examination revealed diffuse iris stromal atrophy with extensive depigmentation and a fixed dilated pupil measuring approximately 4-5 mm (Figure 4).

**Figure 5:**
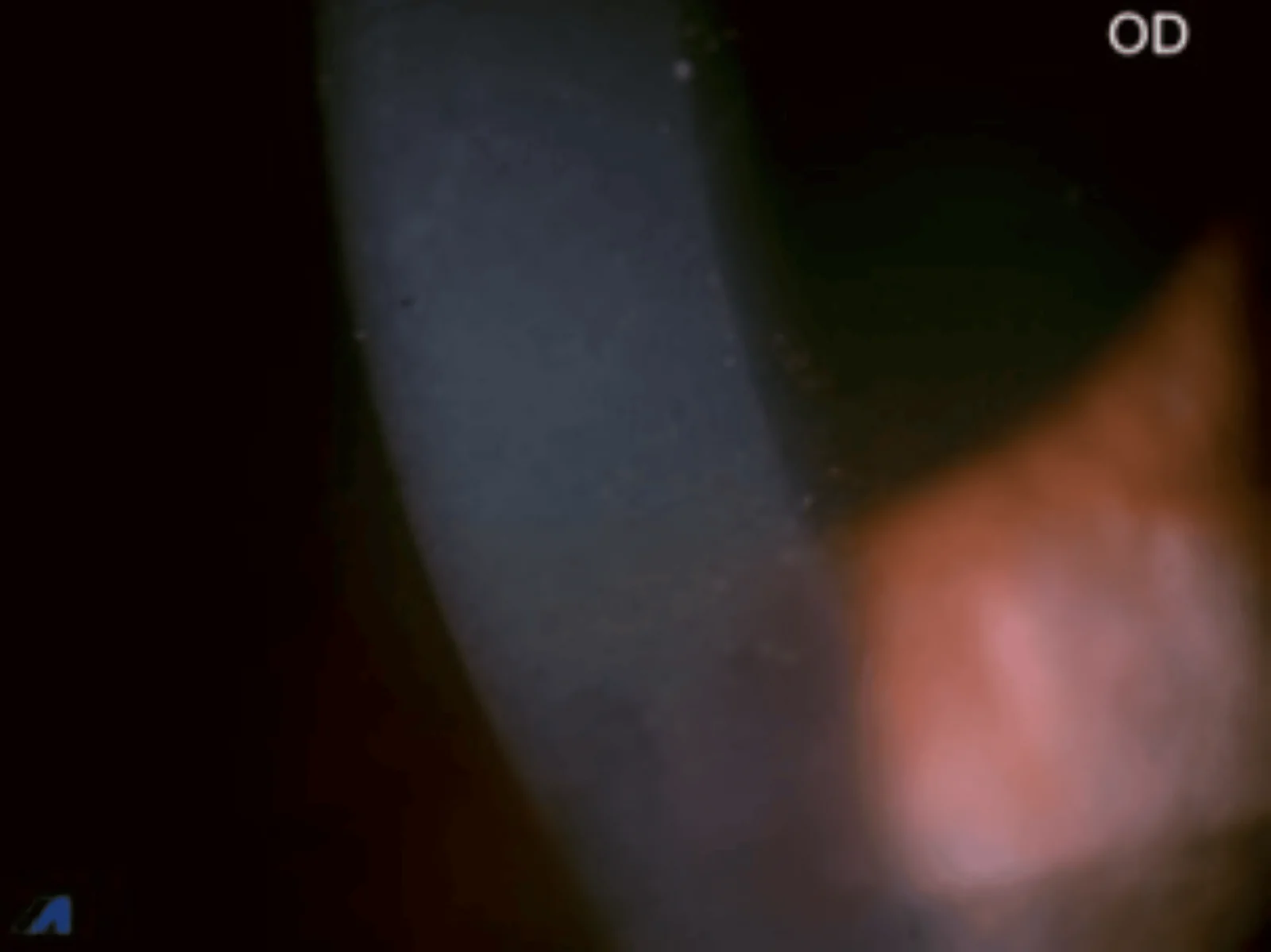
High-magnification slit-lamp image of microbullae on epithelium with posterior corneal surface showing residual endothelial pigment deposition and scattered keratic precipitates after the resolution of acute toxic keratouveitis

High-magnification slit-lamp imaging demonstrated endothelial pigment dusting and residual keratic precipitates over the posterior corneal surface (Figure 5).

**Figure 6:**
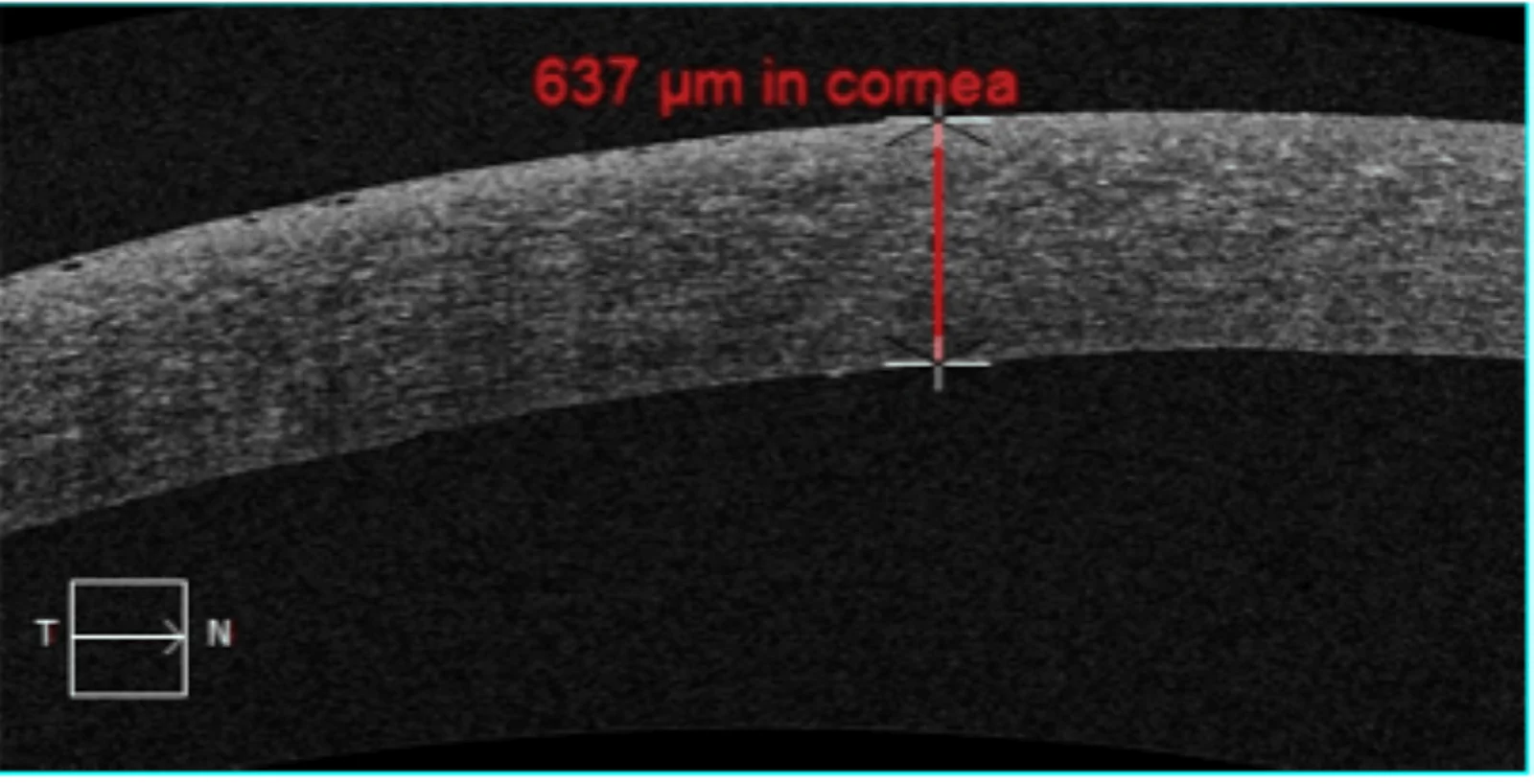
One-month anterior segment optical coherence tomography demonstrating near-normal corneal morphology with minimal residual stromal changes

At the final follow-up at six weeks, BCVA improved remarkably to 6/6 partial. The cornea was largely clear with minimal residual edema and microbullae. The crystalline lens remained clear, and posterior segment examination was unremarkable. Serial anterior segment optical coherence tomography imaging demonstrated progressive restoration of corneal architecture with the resolution of stromal edema (Figure 6).

## Discussion

Honeybee sting injuries to the cornea result from both direct mechanical penetration and toxic venom-induced tissue injury [1,2,4,5]. Melittin, phospholipase A₂, hyaluronidase, and other inflammatory mediators may cause endothelial dysfunction, keratouveitis, cataract formation, secondary glaucoma, optic neuropathy, and permanent visual impairment [2,4,5,8].

In the present case, severe corneal edema, toxic keratouveitis, and markedly elevated intraocular pressure were present at presentation. The retained deep stromal foreign body was associated with surrounding stromal infiltration, suggesting continued local inflammatory activity. Early surgical removal of retained bee sting material likely reduced ongoing venom-mediated toxicity and contributed to the favorable outcome, consistent with previous reports advocating prompt surgical intervention [4,6,7].

A notable clinical finding was the development of diffuse iris stromal atrophy with persistent mydriasis despite the resolution of corneal inflammation. Postoperative slit-lamp photographs demonstrated extensive iris depigmentation, stromal atrophy, endothelial pigment dusting, and residual keratic precipitates. Similar findings have been described following toxic injury to iris tissue and vasculature caused by bee venom components [2,5,8]. Although permanent iris changes persisted, the patient achieved excellent visual recovery.

Serial anterior segment optical coherence tomography imaging documented the gradual restoration of corneal architecture following the removal of the retained sting material and medical therapy. The combination of clinical and imaging findings highlights the importance of multimodal documentation in monitoring recovery following severe ocular bee sting injuries [3-7].

## Conclusions

Deep stromal honeybee sting injury is an uncommon but potentially sight-threatening ocular emergency that may result in severe toxic keratouveitis, corneal endothelial dysfunction, secondary ocular hypertension, iris atrophy, and permanent visual impairment if not recognized and treated promptly. This case highlights the importance of maintaining a high index of suspicion for a retained intracorneal stinger in patients presenting with ocular inflammation following a bee sting. Careful slit-lamp examination, timely removal of the retained foreign body when feasible, and early initiation of intensive anti-inflammatory therapy with appropriate control of intraocular pressure are critical for limiting venom-induced tissue damage and preventing long-term complications. Despite the severity of the initial presentation, favorable anatomical and visual outcomes can be achieved with prompt diagnosis, individualized management, and close follow-up. Reporting such rare cases adds to the limited literature and may help guide clinicians in the evaluation and management of similar injuries in the future.

## References

[1] Gilboa M, Gdal-On M, Zonis S (1977). Bee and wasp stings of the eye. Retained intralenticular wasp sting: a case report. Br J Ophthalmol.

[2] Arcieri ES, França ET, de Oliveria HB, De Abreu Ferreira L, Ferreira MA, Rocha FJ (2002). Ocular lesions arising after stings by hymenopteran insects. Cornea.

[3] Ang WJ, Md Kadir SZ, Fadzillah AJ, Zunaina E (2017). A case series of bee sting keratopathy with different outcomes in Malaysia. Cureus.

[4] Rai RR, Gonzalez-Gonzalez LA, Papakostas TD (2017). Management of corneal bee sting injuries. Semin Ophthalmol.

[5] Gudiseva H, Uddaraju M, Pradhan S, Das M, Mascarenhas J, Srinivasan M, Prajna NV (2018). Ocular manifestations of isolated corneal bee sting injury, management strategies, and clinical outcomes. Indian J Ophthalmol.

[6] Kim JH, Kim M, Lee SJ, Han SB, Hyon JY (2014). Corneal bee sting controlled with early surgical intervention and systemic high-dose steroid therapy. Case Rep Ophthalmol Med.

[7] Razmjoo H, Abtahi MA, Roomizadeh P, Mohammadi Z, Abtahi SH (2011). Management of corneal bee sting. Clin Ophthalmol.

[8] Siddharthan KS, Raghavan A, Revathi R (2014). Clinical features and management of ocular lesions after stings by hymenopteran insects. Indian J Ophthalmol.

